# Penetrating aortic ulcer post migration of thoracic aortic endoprosthesis: Case report

**DOI:** 10.1016/j.ijscr.2021.106219

**Published:** 2021-07-20

**Authors:** E. Dinoto, F. Ferlito, G. Tortomasi, S. Evola, G. Bajardi, F. Pecoraro

**Affiliations:** a Vascular Surgery Unit, AOUP Policlinico ‘P. Giaccone’, Palermo, Italy; b Department of Surgical, Oncological and Oral Sciences, University of Palermo, Italy; c Unit of Cardiology, Department of Health Promotion, Mother and Child Care, Internal Medicine and Medical Specialties (ProMISE) ‘G. D'Alessandro’, University Hospital Paolo Giaccone, University of Palermo, Palermo, Italy

**Keywords:** Complicated aortic B dissection, Penetrating atherosclerotic ulcer, Migration endoprosthesis, TEVAR, Case report

## Abstract

**Introduction:**

Thoracic endovascular aortic repair (TEVAR) is the first treatment option for many thoracic aortic pathologies. Especially after aortic dissections, it is possible to have progression during follow-up with appearance of new lesions on arterial wall. Herein, we report a case of Penetrating Aortic Ulcer (PAU) post release of Thoracic endoprosthesis.

**Presentation of case:**

A 67-years-old male with hypertension and diabetes mellitus was followed at our hospital after an emergency procedure for Type B aortic dissection (TBAD) complicated by symptomatic large infrarenal AAA and treated with a proximal TEVAR plus chimney for left subclavian artery and PETTICOAT with EVAR for abdominal aortic disease. Follow up at 15 months showed a deep PAU with partial crush of stent in Left Subclavian Artery. Thus, we performed a left carotid-subclavian bypass and subsequently a TEVAR procedure with release of Bolton Relay endoprosthesis (Terumo Aortic, Sunrise, Florida, United States).

**Discussion:**

In literature there are few studeis that focus on migration after TEVAR during follow-up. Elongation, changes of tortuosity on thoracic aorta after TEVAR, can help to determine a migration of prosthesis. In this case Bolton Relay endoprosthesis (Terumo Aortic, Sunrise, Florida, United States) has permitted to improve precision and quality of procedure.

**Conclusion:**

In literature there are few studies reporting complications of TEVAR post prosthesis migration. In this case, Bolton Relay endoprosthesis was useful and safe.

## Highlights

• TEVAR is the first treatment option for many thoracic aortic pathologies.
• In literature there are few study that focus on migration after TEVAR.
• Changes on thoracic aorta can help to determine a migration of prosthesis.
• PAU after TEVAR is a rare complication.
• Bolton Relay Thoracic Graft allowed the treatment of PAU in complicated landing zone.

## 1 Introduction

Over the last decade, thoracic endovascular aortic repair (TEVAR) has evolved as the first-line treatment option for many thoracic aortic pathologies due to reduced perioperative morbidity and mortality rates in selected patients [1]. Two important anatomical features can sometimes impede the applicability of this treatment in the proximal thoracic aorta. First, the supra-aortic branches limit the proximal extension of the stent-graft. Second, a Gothic aortic arch configuration that is a sharply and angulated aortic arch. This may significantly impede advancement, precise placement and deployment of the device [2]. A significant proportion (12%–60%) of patients, especially with aortic dissections, may have experience disease progression during follow-up after TEVAR, and thus requires further surgical procedures [3].

Herein, we report a case of Penetrating Aortic Ulcer (PAU) post release of Thoracic endoprosthesis in a case of complicated type B aortic dissection (TBAD).

This work has been written in accordance with the SCARE criteria [4].

## 2 Case report

**Fig. 1:**
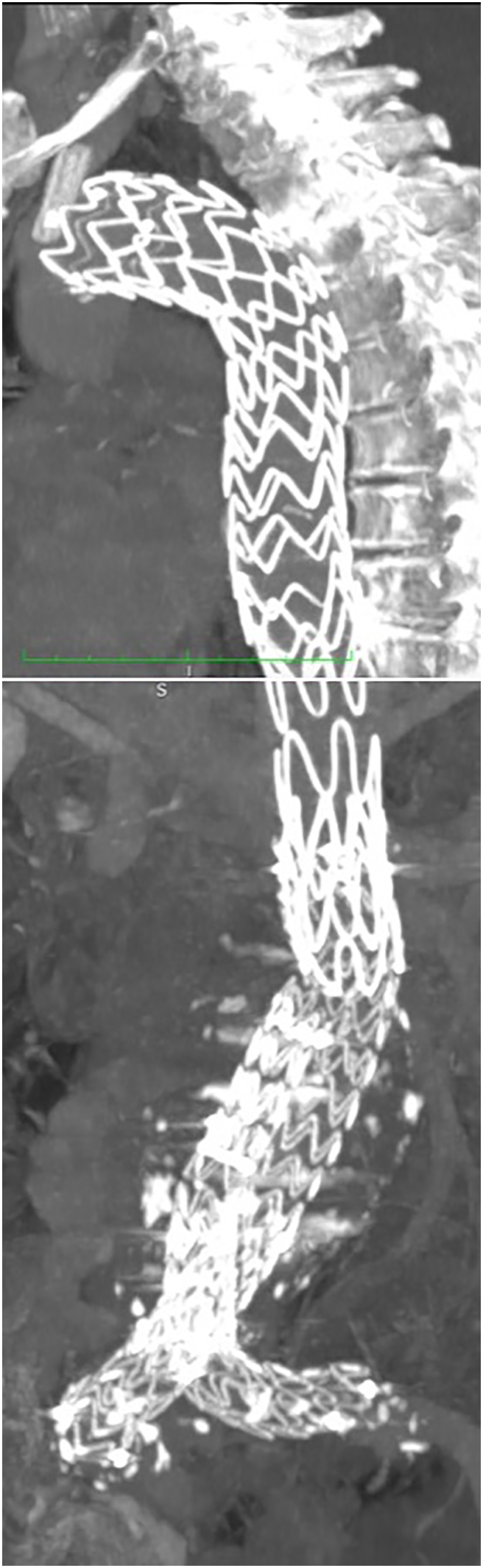
Post-operative CT MPR after the first procedure showing Thoracic graft (A) and abdominal graft (B).

**Fig. 2:**
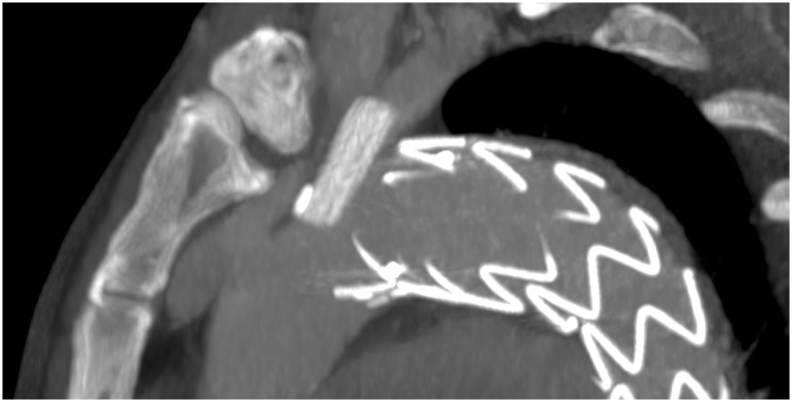
Post-operative CT-angiography after the first procedure showing the good positioning of Thoracic graft and stent in left subclavian artery.

A 67-years-old male with hypertension and diabetes mellitus was followed at our hospital after an emergency procedure for TBAD complicated by symptomatic large infrarenal AAA. Patient was treated 18 months before in the same session with TEVAR releasing in thoracic aorta a 40 × 223 mm Valiant Navion (Medtronic, Inc., Minneapolis, MN, USA) and performing a PETTICOAT (provisional extension to induce complete attachment technique) with a 36x180mm Zenith endovascular dissection stent (Cook Medical, Bloomington, IN, USA). To treat a symptomatic AAA was released a 32 mm bifurcated ENDURANT II stent-graft (Medtronic, Inc.) (Fig. 1). In order to preserve flow on the left subclavian artery (LSA), a chimney technique was performed with 10 × 31 mm Silene covered stent (InSitu Technologies Inc., St. Paul, MN, USA). CT angiography check, during the first hospitalization, showed good placement of thoracic and abdominal graft with complete exclusion of AAA and patency of LSA in absence of endoleak (Fig. 2).

**Fig. 3:**
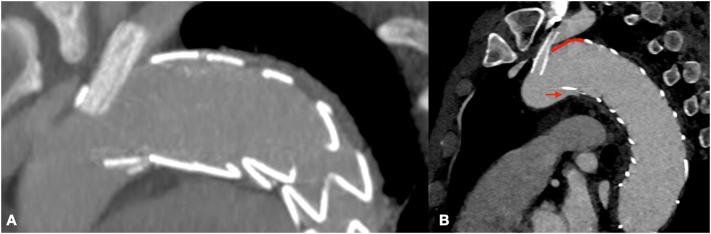
Post-operative CT-angiography after the first procedure (A) and Follow-up at 12 months showing migration of prosthesis and penetrating aortic ulcer (B).

**Fig. 4:**
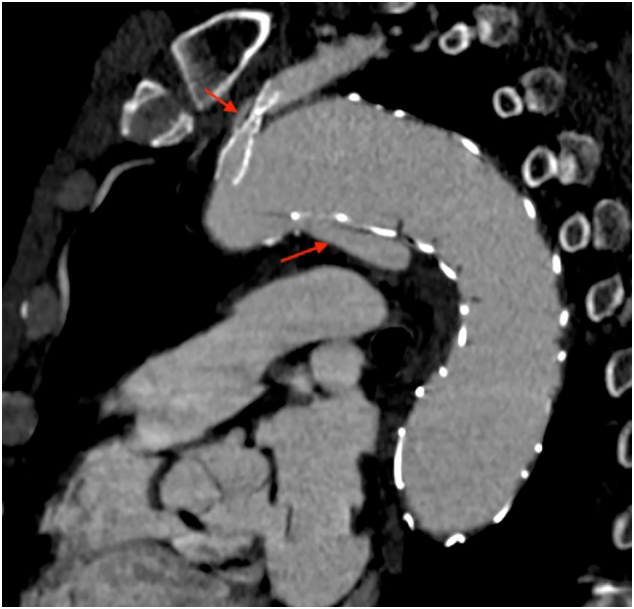
Follow-up 15 months CT-angiography showing deterioration of penetrating aortic ulcer and crashing of stent in left subclavian artery.

**Fig. 5:**
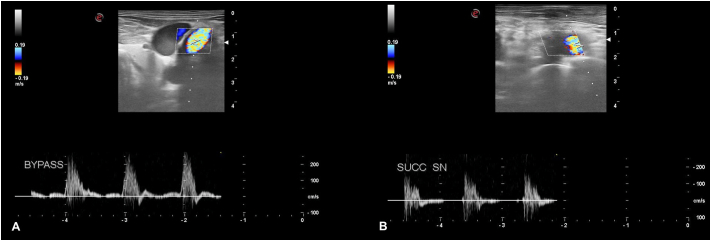
Ultrasound doppler showing patency of Bypass (A) and left carotid-subclavian bypass (B).

Follow up at 12 months, after CT-scan, showed a PAU on little curve of aortic arch with migration of prosthesis (Fig. 3). Follow up at 15 months showed an increase of PAU with partial crush of stent in LSA without significative change of flow in left arm (Fig. 4). Thus, to prevent further deterioration of aortic lesion, we chose to have a second procedure. The distance from Left Common carotid artery (LCC) to proximal end of prosthesis was 10 mm and in order to increase length of landing zone was necessary to cover LSA and previous chimney. To avoid an occlusion of LSA, was performed a first step with left catotid - subclavian bypass (Fig. 5) and a second procedure with release of Bolton Relay endoprosthesis (Terumo Aortic, Sunrise, Florida, United States).

Intervention was conducted without placement of spinal drain.

**Fig. 6:**
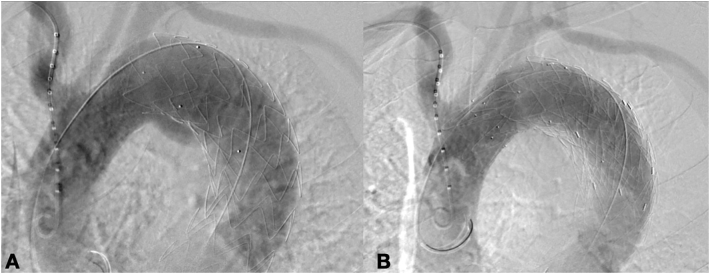
Intraoperative Angiography before release of Bolton Relay Graft (A) and final outcome (B).

Under general anesthesia and systemic heparinization (ACT >250 s), a right surgical common femoral artery (CFA) and a right percutaneous brachial artery (BA) accesses were gained. A Bolton Relay PRO 38 × 150 mm and Bolton Relay NBS 42 × 100 mm was placed and deployed in zone 2 aortic arch in front of the proximal end of Navion. The control angiography confirmed the adequate proximal sealing, the absence of leakages and LSA patency (Fig. 6).

After procedure the patient was transferred in the intensive care unit (ICU) for monitoring of vital functions where the extubation was carried after 12 h with no complications or signs of spinal cord ischemia (SCI). On the second postoperative day the patient was transferred from ICU and discharged at home after seven days.

## 3 Discussion

In literature there are few studies that focus on migration after TEVAR during follow-up. Geisbüsch P. et Al. report a migration endoprosthesis rate of 7.3%. In this series, all migrations occurred in patients with TAA and TBAD, with a statistically significant increased risk for migration in patients with TAA. Both diseases are frequently characterized by pathologic changes of the entire thoracic aorta that can lead to elongation, changes of tortuosity, and loss of primary fixation points, thus contributing to graft migration [3], [5]. We suppose that migration of endoprosthesis together with fragility of aortic wall were the causes of PAU. Pitton M.B. et Al report a series where after TEVAR, secondary interventions with stent-graft extensions were performed for aortic wall ulcers caused by bare stent struts [6]. A problematic landing zone in aortic arch can be approached by special thoracic grafts [7]. These, originally developed from stent grafts designed for the infrarenal aorta, were undergone successive iterations to become more well-suited to the specific anatomic challenges presented by the thoracic aorta [8]. The Bolton Relay Thoracic Stent Graft with Plus Delivery System (Bolton Medical, Sunrise, Fla) is a modular device with curved nitinol wire providing flexibility, torque response, and columnar support, was designed specifically to handle the curvature and tortuosity of the thoracic aorta [9]. The RelayPlus Delivery System consists of a series of coaxially arranged sheaths and catheters, along with a tubular handle control system. The delivery mechanism consists of two stages. First, a hydrophilically coated introducer (Outer Primary Sheath), is used to advance and track over a guidewire. The tip of the Outer Primary Sheath contains a preformed curve. Within the first stage is the second stage. Second, a flexible sheath in which the stent-graft is compressed. The flexibility of the second stage permits tracking through tortuous and curved portions of the thoracic aorta. The stent-graft is self-expandable and the delivery system is withdrawn after deployment. In this case reported Bolton Relay Thoracic Stent Graft (Bolton Medical, Sunrise, Fla) has permitted to have a controlled release, improving precision and quality of procedure.

## 4 Conclusions

In literature there are few studies reporting complications of TEVAR post prosthesis migration. PAU after TEVAR is a rare complication. Also in our experience, the use of Bolton Relay Thoracic Stent Graft (Bolton Medical, Sunrise, Fla) was safe and technically feasible and allowed the treatment of PAU placed in complicated landing zone.

## Funding

None.

## Ethical approval

None.

## Consent

Not applicable.

## Author contribution

Ettore Dinoto: study concept, design, data collection, data analysis, interpretation, writing the paper, final approval of the version to be submitted, guarantor.

Felice Pecoraro: study concept, design, data collection, data analysis, interpretation, writing the paper, final approval of the version to be submitted.

Ferlito Francesca: study concept, design, data collection, data analysis, interpretation, final approval of the version to be submitted.

Tortomasi Graziella: study concept, design, data collection, final approval of the version to be submitted.

Evola Salvatore: study concept, design, data collection, final approval of the version to be submitted.

Guido Bajardi: study concept, design, data collection, data analysis, interpretation, final approval of the version to be submitted.

## Provenance and peer review

Not commissioned, externally peer-reviewed.

## Registration of research studies

Not applicable.

## Guarantor

Ettore dinoto.

## Declaration of competing interest

None.
